# Effect of Probiotics Supplementation on Cortisol Levels: A Systematic Review and Meta-Analysis

**DOI:** 10.3390/nu16203564

**Published:** 2024-10-21

**Authors:** Manav Jain, Aishwarya Anand, Nisha Sharma, Muhammad Aaqib Shamim, Elena Y. Enioutina

**Affiliations:** 1 Division of Clinical Pharmacology, Department of Pediatrics, Spencer Fox Eccles School of Medicine, University of Utah, Salt Lake City, UT 84108, USA; manav.jain@hsc.utah.edu; 2 Department of Pathology, University of Maryland School of Medicine, Baltimore, MD 21201, USA; niehaaa@gmail.com; 3 Department of Pharmacology, Postgraduate Institute of Medical Education and Research, Chandigarh 160012, India; nishasharma.nics8@gmail.com; 4 Department of Pharmacology, All India Institute of Medical Sciences, Jodhpur 342005, India; aaqibsh@gmail.com

**Keywords:** probiotics, cortisol, gut-microbiota, lactobacillus, bifidobacterium

## Abstract

**Background**: Several randomized controlled trials (RCTs) have shown conflicting results on cortisol levels following probiotic administration in healthy and diseased populations. Previous analyses were inconclusive due to limited studies, and evidence is lacking on how these effects vary by health status; region; therapy duration; medications, and use of single or multiple strains. **Methods**: In this systematic review and meta-analysis (PROSPERO [CRD42024538539]), we searched PubMed, Cochrane Library, Embase, Scopus, Web of Science, CINAHL, ProQuest, and Web of Science Preprints until 13 August 2024, for RCTs on probiotic administration, either alone or combined, across all age groups and without specific medical condition requirements. We applied random-effects meta-analysis, assessed bias using the Cochrane RoB 2 tool, and evaluated evidence certainty with GRADE. **Findings**: We screened 1739 records and retrieved 46 RCTs (3516 participants). Probiotics supplementation decreased cortisol levels compared to the control arm [46 RCTs; SMD: −0.45; 95% CI: −0.83; −0.07; *I*^2^: 92.5%, low certainty]. Among various subgroups; probiotics supplementation decreased the cortisol levels in the subgroups without concomitant medications [37 RCTs; SMD: −0.30; 95% CI [−0.58; −0.03], *I*^2^: 88.7%] with a single probiotic strain [30 RCTs; SMD: −0.33; 95% CI: −0.63; −0.028; *I*^2^: 88.8%], in a healthy population [35 RCTs; SMD:−0.3; 95% CI: −0.58; −0.03; *I*^2^: 88.7] and in the Asia region [21 RCTs; SMD: −0.83; 95% CI: −1.58; −0.07; *I*^2^: 95%]. **Interpretation**: A low level of evidence suggests probiotics might reduce cortisol levels, but more targeted studies are needed to identify variables affecting the response in specific subgroups.

## 1. Introduction

Activation of the hypothalamic–pituitary–adrenal (HPA) axis is the main component of the body’s response to stress, producing and releasing a glucocorticoid hormone—cortisol—to maintain homeostasis. Cortisol affects various physiological processes, such as the functioning of the immune system, metabolism, and stress response. Increased activity of the HPA axis and elevated cortisol levels have been associated with various disorders such as anxiety, depression, and metabolic syndrome [1,2].

There is growing interest in understanding how the gut–brain axis influences brain function and human responses. The gut–brain axis is a bidirectional communication between the gut microbiota (GM) and the HPA axis connecting the gastrointestinal tract and the central nervous system [3], with HPA playing a crucial role. There is evidence that cortisol directly affects gut function since cortisol receptors are expressed on various gut cells, including immune cells, enteroendocrine cells, and epithelial cells [4,5]. In addition, cortisol can modify intestinal permeability, gastrointestinal transit time, and nutritional availability, all of which can impact the variety and composition of the gut microbiota [5]. Moreover, cortisol can affect the brain by binding to glucocorticoid receptors (GRs) in the prefrontal cortex, amygdala, and hippocampal areas. Since bacteria in the gut can trigger stress circuits in the brain through the vagus nerve and sensory neurons in the enteric nervous system, there is more evidence supporting signaling between the GM and CNS [6].

Research has shown that gut microbiota may influence brain function through multiple mechanisms, including immune response modulation, neurotransmitter production, and direct effects on the HPA axis [6,7]. In this regard, probiotics have drawn interest because of their capacity to modify the HPA axis and impact cortisol production. Probiotics regulate the microecological balance of the intestine and can be helpful when used in adequate doses [8]. Probiotics have been explored as a potential target in various conditions like depression [9], anxiety [10], polycystic ovarian syndrome [11], autism spectrum disorder [12], cognitive impairment [13], obesity [14,15], and stress [16]. They act by affecting the levels of various bioactive substances, including cortisol.

Several randomized control trials have assessed cortisol levels in both healthy and diseased populations following probiotic administration. The studies showed conflicting results. Most studies evaluating cortisol levels in healthy individuals with stress found no significant difference in cortisol levels between the probiotic and control groups; however, a few studies reported significantly higher cortisol levels after exam stress in the intervention group [17,18]. Shi et al. found lower levels of cortisol in adolescent patients with major depression and suggested that cognition could be improved in these patients by regulating cortisol [19]. Cortisol levels are known to be higher in patients with Alzheimer’s disease (AD) and are believed to be a risk factor for cognitive degeneration. However, administration of muti-strain probiotic supplements did not show significant changes during the intervention [20].

A previous meta-analysis by Sikorska et al. found no significant difference in cortisol levels in a subgroup after probiotic administration between the intervention and control group in patients with depression [9]. Similarly, Zhang et al. concluded that although probiotic administration reduced the subjective level of stress and improved stress-related anxiety/depression levels, the effect on cortisol levels was not significant [16]. However, a limited number of studies in previous analyses rendered the results inconclusive.

There is a need for a comprehensive review to determine whether probiotics administration affects cortisol levels and how this effect varies with health status (diseased or healthy), geographical region, duration of therapy, use of concomitant medications, and number of strains administered. This review will generate evidence-based information on the relationship between the administration of probiotics and cortisol levels.

## 2. Methods

### 2.1. Protocol Registration and Reporting

The protocol is registered at PROSPERO (CRD42024538539). This systematic review and meta-analysis (SRMA) followed Preferred Reporting Items for Systematic Reviews and Meta-analysis (PRISMA) 2020 guidelines [21].

### 2.2. Research Question and Selection Criteria

This SRMA assessed whether probiotics administration decreased cortisol levels in the participants. Studies meeting all the following criteria are considered suitable for inclusion: **1.** Population: Participants across all age groups with no specific medical conditions or disorders; **2.** Intervention: Probiotics were given as a standalone or part of a combination therapy; **3.** Control: Placebo, routine care, or any active comparator other than probiotics; **4.** Outcome: Plasma, serum, salivary, urinary, or fecal cortisol (wherever multiple samples were available, we chose plasma cortisol); **5.** Timepoint: Samples analyzed just before or after the time point of maximum stress were included for the studies evaluating stress. Samples taken within or at 30 min after waking up in the case of morning samples, samples measuring Cmax, and samples measured at a single time point were also included; **6.** Study design: RCT only. Detailed selection criteria are mentioned in the supplementary **[Table S1]**.

### 2.3. Systematic Search and Data Extraction

We systematically searched PubMed, Cochrane Library, Embase, Scopus, Web of Science (WoS), CINAHL, ProQuest, and WoS Preprints until 13 August 2024. MJ and EE prepared a list of key concepts, synonyms, and acronyms. MJ prepared a database-specific search strategy using MeSH/Emtree terms, truncated terms, and keywords **[Table S2]**. EE peer-reviewed this per the Peer Review of Electronic Search Strategies checklist [22]. We checked references of selected articles and conducted a forward citation search to identify additional articles. After the search and deduplication, two authors (amongst MJ, AA, and NS) screened the records and extracted data. In case of any disagreement, MJ and EE resolved the conflict by discussing it among themselves. Data extraction used a spreadsheet containing the authors’ name and study year, number of strains used, type of strains administered, health condition evaluated, study region, total number of participants, body mass index (BMI), mean age in probiotics and control group, use of concomitant medications or not, and outcome data.

### 2.4. Statistical Analysis

#### 2.4.1. Synthesis

The continuous data was extracted as Mean ± SD or Mean (95% CI) or Median (IQR) or Median (range). The values of the studies that reported only changes from baseline data were included. All the data were transformed into Mean ± SD (https://www.math.hkbu.edu.hk/~tongt/papers/median2mean.html [Accessed on 20 August 2024]). Data from the plot was extracted, wherever needed, using WebPlotDigitizer v5.0 (https://automeris.io/wpd/ [Accessed on 20 August 2024]). For the missing values in the continuous predictor variables, we used the median imputation method for total males and females, and we maintained the proportion according to each study’s information. To address the unit of analysis issue, we expressed data as standardized mean difference (SMD) with a 95% confidence interval. To avoid the overestimation of precision due to the double counting of groups in the studies having more than 2 groups, we pooled data from the groups receiving the probiotics and compared them with the control [23]. We employed a random-effects model for synthesis due to the clinical between-study heterogeneity. We used Knapp–Hartnup adjustments to calculate CI around the pooled effect. We also provided the 95% prediction interval (95% PI) [24]. The pooled estimate was expressed using forest and drapery plots [25]. We also included the details of the risk of bias assessment and certainty in evidence alongside details regarding meta-analytical methods in the plots for comprehensiveness and quicker interpretation.

#### 2.4.2. Heterogeneity and Sensitivity Analyses

To assess heterogeneity, we used I-squared (*I*^2^), Q-test, tau, and tau-squared values to avoid reliance on a single metric [23]. We used I-squared and a Q-test to represent the proportion of heterogeneity attributed to between-study heterogeneity. We used the Sidik–Jonkman estimator to estimate the tau-squared and the Q-profile method to determine the confidence interval of the tau-squared. To explore heterogeneity, we first performed subgroup analyses based on population (diseased or healthy), treatment duration (≤6 or >6 weeks), number of strains (≤1 or >1), presence or absence of concomitant medications, and region. For analysis purposes, we considered stress to be healthy. To address any heterogeneity that remained after subgroup analysis, we performed meta-regression using covariates like treatment duration, number of strains administered, and total number of males and females. For sensitivity analysis, we performed a leave-one-out meta-analysis and outlier detection. We used contour-enhanced funnel plots and Egger’s regression (threshold of *p* < 0.1) to detect publication bias [26]. All the analyses were performed using meta, metafor, metasens, and brms packages in R software (v4.3.0) [27].

#### 2.4.3. Quality and Evidence Certainty Assessment

Two of the authors performed these steps. They evaluated the quality of the selected studies for individual eligible outcomes, as recommended by the Cochrane Risk of Bias version 2.0 tool (RoB 2 tool) [28]. A judgment of low risk of bias, some concerns, or high risk of bias was assigned to each domain and to the overall result. We used the GRADE (grading of recommendations assessment, development, and evaluation) methodology to assess the certainty of the synthesized evidence and reported it in the forest plot [29].

## 3. Results

### 3.1. Study Selection and Characteristics

**Figure 1:**
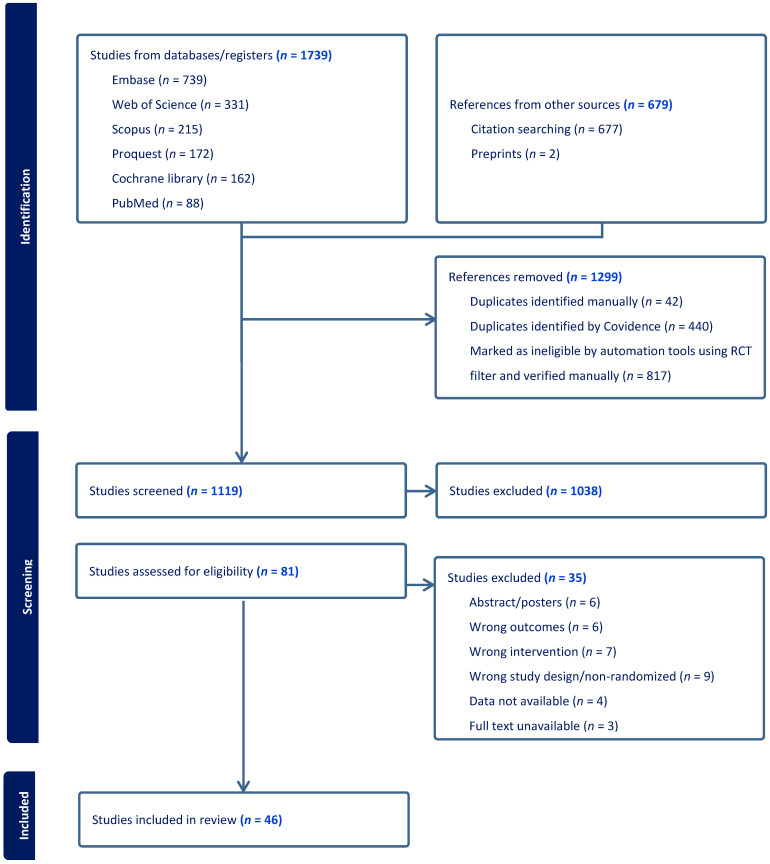
PRISMA flow chart.

We identified 46 RCTs with 3516 participants [17,18,19,20,30,31,32,33,34,35,36,37,38,39,40,41,42,43,44,45,46,47,48,49,50,51,52,53,54,55,56,57,58,59,60,61,62,63,64,65,66,67,68,69,70,71] **[Figure 1]**. We excluded several studies due to different reasons **[Table S3]**. Of all the studies, 21 were conducted in Europe (EU) and Asia, 3 in the USA, and 1 in Africa. A total of 21 studies recruited participants from the community/databases, 14 from universities, 10 from hospitals, and 1 from a school. The number of strains and treatment duration administered varied in studies. The number of strains ranged from 1 to 14, while the median (range) treatment duration was 7.21 (1–24) weeks **[Table S4]**.

### 3.2. Pooled Estimate

**Figure 2:**
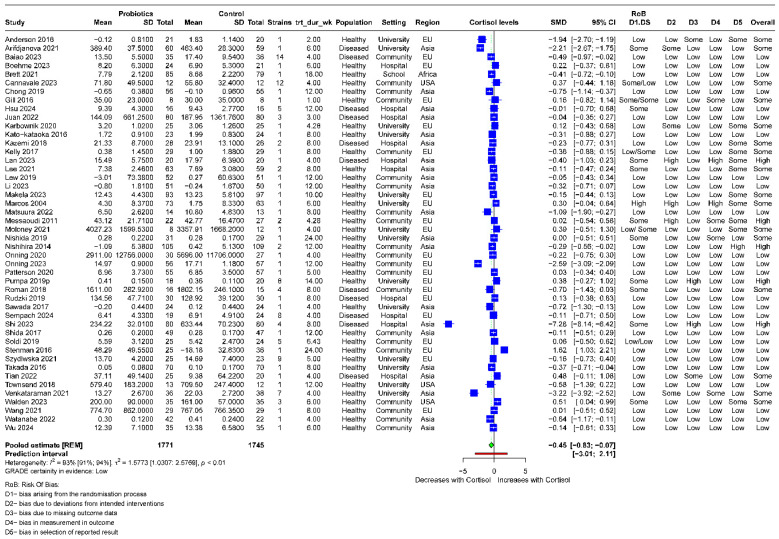
Forest plot showing pooled estimates in a complete case analysis(D1–D5—domains for RoB assessment in parallel group design studies, DS—additional domain besides D1–D5 in cross-over design studies).

After pooling data from all the studies, our results showed that probiotics supplementation decreased the levels of cortisol compared to the control arm [46 RCTs, SMD: −0.45; 95% CI: −0.83, −0.07; *I*^2^: 92.5%, *p*: <0.0001] **[Figure 2]**.

### 3.3. Subgroup Analysis and Meta-Regression

Subgroup analyses in predefined categories were not able to address the heterogeneity, although, among various subgroups, probiotics supplementation decreased the cortisol levels in the subgroups without concomitant medications, with a single probiotic strain, in a healthy population and in the Asia region **[Table 1]**. Within each subgroup category, the groups were not significantly different from each other. Meta-regression using the treatment duration, number of strains administered, and total number of males and females could account for only 18.42% of heterogeneity **[Table S5]**. We could not use age and BMI due to the large number of missing values.

### 3.4. Sensitivity Analysis and Outlier Detection

**Figure 3:**
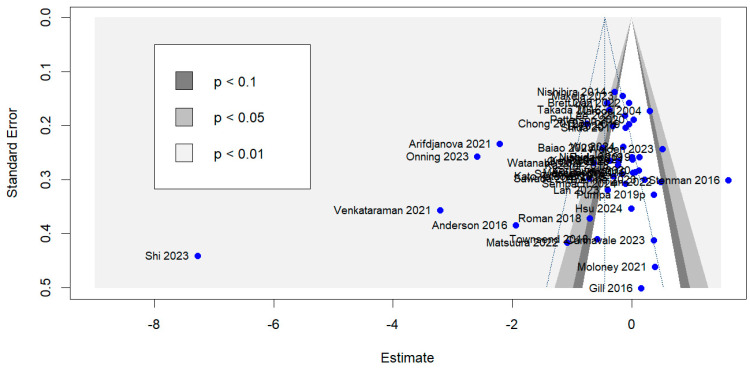
Funnel plot with complete cases.

**Figure 4:**
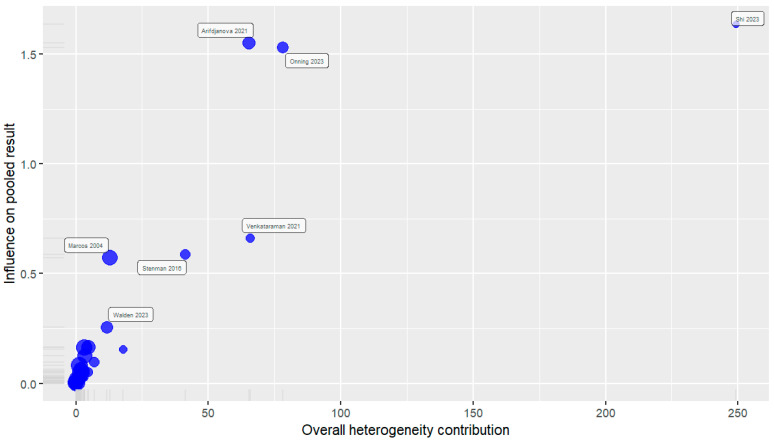
Baujat plot showing outliers.

We conducted a sensitivity analysis by omitting each study once for the outcome and the estimated pooled effect called the leave-one-out meta-analysis. The analysis showed that there was no undue influence of any single study on the pooled outcome **[Figure S7]**. Based on the visual asymmetry in the funnel plot **[Figure 3]** and heterogeneity contribution and influence on pooled results in the Baujat plot **[Figure 4]** by individual studies in outlier detection, we found eight studies distorting the pooled effects and on removal of these studies, we found significant reduction in the heterogeneity [38 RCTs, SMD: −0.18, 95% CI: −0.28; −0.08, *I*^2^: 26.4%, *p*: 0.07] **[Figure S8]**.

### 3.5. Risk of Bias (RoB), Publication Bias, and GRADE Assessment

Although most of the included studies had an overall low risk of bias in each domain, only 25 studies had a low risk of bias, while 16 had some concerns and 5 had a high risk **[Figure S9]**. The outcome-wise risk of bias assessments for each study contributing to each outcome is given in the individual forest plots for better interpretation. We did find asymmetry visually (inspection of the funnel plot) in the contour-enhanced funnel plot and statistically (Egger’s regression test, *p* = 0.29) after outlier removal **[Figure S10]**. Based on the GRADE assessment, the certainty of the evidence was low in complete case analysis and moderate after outlier removal **[Table S6]**.

## 4. Discussion

This SRMA of 46 RCTs involving 3516 participants assessed the impact of probiotics supplementation on cortisol levels. The analysis included diverse geographical regions, age groups, health conditions, treatment durations, and probiotic strain combinations. The pooled estimates showed that probiotics significantly reduced the cortisol levels compared to the control group; although there was substantial heterogeneity [*I*^2^ = 92.8%, *p* < 0.0001], indicating wide differences among the included studies.

Subgroup analyses help us to identify why a specific pattern of heterogeneity exists in data. Subgroups should be specified as a priori. We hypothesized that the number of probiotic strains, treatment duration, health status, use of concomitant medication, and geographical region could affect the outcome. It is worth noting that studies from Asia [17,19,20,30,31,35,36,40,42,49,50,53,56,57,58,59,61,62,63,65,67,70], studies conducted without concurrent medication [17,18,30,31,33,34,36,37,38,40,42,44,45,46,47,48,49,50,52,53,54,55,56,57,58,60,62,64,65,66,67,68,69,71], studies administering a single probiotic strain [18,31,32,34,35,36,38,40,42,43,46,47,48,50,51,53,54,55,56,57,59,60,62,64,65,67,68,71], and studies with healthy populations [17,18,30,31,32,33,34,36,37,38,39,40,42,45,46,47,48,49,50,51,52,53,54,55,56,57,58,60,62,63,64,65,66,67,68,71] demonstrated a significant decrease in cortisol levels. These groupings did not significantly differ from one another, even though there was substantial heterogeneity within each category. The treatment duration did not affect the outcome of our analysis. Similar results were found in earlier assessments of BDNF levels, which showed that treatment duration had no effect on BDNF levels and that studies carried out in Asia had shown increases in BDNF levels [72]. Similar to BDNF findings, we expected multistrain probiotics to significantly affect cortisol levels more than single-strain probiotics; however, these variations may be explained by potential interactions among strains [72]. The use of concomitant medications also might have interacted with the probiotic strains.

To investigate possible sources of heterogeneity further, meta-regression was performed using covariates like the total number of males and females, number of strains, and length of treatment. Despite these efforts, it could not explain heterogeneity, indicating that the observed variability may be caused by additional unmeasured factors. The exclusion of age and BMI due to missing data might have limited the explanatory power of the meta-regression.

Sensitivity analysis was performed to evaluate the robustness of the findings, and it was found that the pooled estimate was not disproportionately affected by any one study. Eight studies, however, were shown to have substantially affected the total effect magnitude through outlier detection [17,19,33,38,47,54,70,71]. Following the removal of these outliers, the effect size was somewhat diminished, and the heterogeneity was significantly reduced. Also, the 95% CI around the pooled estimate narrowed. This suggests that the initial heterogeneity was driven, in part, by a few outlier studies. These studies did not significantly differ from the others regarding covariates that could potentially cause heterogeneity, except for two studies: one conducted with adolescents [19] and the other with young adults [17]. Although exploratory subgroup analysis based on the risk of bias could not explain the observed heterogeneity, the studies with high risk and some concerns had more heterogeneity and wider CI as anticipated. Also, the certainty of evidence was moderate, which suggested that, although the results were robust, the unexplainable heterogeneity could not be addressed in our analysis.

None of the previous analyses assessed cortisol levels after probiotic administration. Only two analyses previously assessed cortisol levels after probiotic administration. Zhang et al. evaluated cortisol as a secondary outcome for the assessment of stress in healthy individuals and found no difference in cortisol levels [2 RCTs, SMD: −0.02, 95% CI: −0.34; 0.30, *I*^2^: 0, *p*: 0.89] [16]. In our analysis, we found significant differences in cortisol levels among healthy individuals experiencing varying levels of stress [35 RCTs; SMD: −0.3, 95% CI: −0.58, −0.0002, *I*^2^: 87.4]. We included studies evaluating various types of stress, including academic examinations, competition, and exercise-induced stress, and which used various stress tests, to provide a more generalized effect of probiotics on cortisol. Sikorska et al. analyzed the reduction in cortisol levels as one of the molecular mechanisms of probiotics in depression. The findings showed that the addition of probiotics did not change the cortisol levels compared to the control [3 RCTs, SMD: −0.1, 95% CI: −0.36, 0.15, *I*^2^: 0, *p*: 0.11] [9]. Our exploratory analysis in patients with depression agreed with the previous findings and found no reduction in the level of cortisol [6RCTs, 402 participants, SMD: −0.34, 95% CI: −1.4; 0.71, *I*^2^: 94%, *p*: <0.0001]. Including a larger number of studies in our analysis provided more robust evidence in both settings.

The HPA is crucial in mediating stress responses and regulating the interactions between the gut, gut microbiota, and brain. Prolonged or persistent stress can cause the HPA axis to become dysregulated, which can negatively impact various body systems, including the gut microbiota axis. High cortisol levels have been linked to changes in the composition of gut microbiota and increased permeability of the gut, which can cause inflammation and worsen a variety of CNS illnesses as well as brain dysfunction [6]. In Alzheimer’s disease, cortisol is linked to cognitive decline, and lowering cortisol levels is expected to improve cognition. In the studies assessing the effect of probiotics on cognition, cortisol levels remained unchanged between the groups, although cognition was evaluated under different health conditions [20,44,63]. Stress is considered one of the major risk factors in the development of major depressive disorders [73]. Elevated levels of cortisol, corticotrophin-releasing hormone (CRH), adrenocorticotrophic hormone (ACTH), and pro-inflammatory cytokines were reported in major depressive disorder patients [74]. Most clinical trials showed no reduction in the cortisol levels in patients with depression, except in major depression in adolescents and mild depression in adults where cortisol levels were decreased [33,35,41,61,69,71]. The trials varied in number and type of strains, treatment duration, age, and disease severity, which might have affected the outcome in different studies, although the other confounders cannot be ignored. Lan et al., while evaluating the connection between cortisol and sleep quality, suggested some regulatory effects of the probiotic strain Bifidobacterium breve CCFM105 on the HPA axis, but overall found no difference in cortisol levels at the end of the intervention [59]. Overall, in this review, we did not find significant differences in the levels of cortisol after probiotics administration, irrespective of the type of disease condition.

Although this is one of the most exhaustive analyses to examine the effect of probiotics on cortisol levels, this study also has its limitations. We could address heterogeneity only by outlier detection based on visual asymmetry in funnel plots and heterogeneity contribution and influence on pooled results. It is still considered as a brute-force approach. We could not use age and BMI as predictors to assess heterogeneity as there was a lot of missing data. Also, we could not use the type of strains and dose of each probiotic in the analysis as it became too heterogeneous to conduct a meaningful analysis. For assessing heterogeneity, we used prediction intervals along with other measures. Since it included zero, we were less sure about our findings, and future studies might not reduce cortisol levels. Diet is known to affect gut microbiota, and the effect of diet cannot be ignored.

## 5. Conclusions

This systematic review and meta-analysis showed that supplementation with probiotics may reduce cortisol levels, as evident by low to moderate certainty of evidence. The effect was more evident in certain subgroups. The heterogeneity, however, could not be adequately explained, indicating that additional, unmeasured factors might influence the results. The results highlight the promise of probiotics in treating stress-related disorders; nevertheless, more research is required to elucidate the underlying mechanisms and identify variables affecting the response.

## Figures and Tables

**Table 1:** Subgroup analysis for the effects of probiotics on cortisol levels.

Subgroups (N)	Random Effects SMD [95% CI]	*p*_subgroup_	*I*^2^ (%)	Additional Information
**Strains**				**[Figure S1]**
Equal to 1 (30)	**−0.33 [−0.63; −0.028]**		88.8	
More than 1 (16)	−0.69 [−1.72; 0.33]	0.46	95.6	
**Treatment duration (weeks)**				**[Figure S2]**
Less than or equal to 6 (22)	−0.34 [−0.76; 0.07]	0.59	89.7	
More than 6 (24)	−0.55 [−1.21; 0.11]		94.2	
**Population**				**[Figure S3]**
Diseased (11)	−0.97 [−2.44; 0.49]	0.31	96.9	
Healthy (35)	**−0.29 [−0.58; −0.0002]**		87.4	
**Concomitant medication**				**[Figure S4]**
Yes (9)	−1.06 [−2.93; 0.81]	0.36	97.5	
No (37)	**−0.30 [−0.58; −0.03]**		88.7	
**Region**				**[Figure S5]**
Asia (21)	**−0.83 [−1.58; −0.07]**	0.17	95.0	
EU (21)	−0.16 [−0.54; 0.22]		88.1	
US (3)	0.15 [−1.3; 1.6]		62.7	
Africa (1)	**−0.40 [−0.72; −0.1]**		-	
**Risk of bias (RoB)**				**[Figure S6]**
Low (25)	−0.28 [−0.56; 0.001]	0.65	84.3	
Some concerns (16)	−0.41 [−0.99; 0.17]		92.3	
High (5)	−1.5 [−5.51; 2.5]		98.4	

## Data Availability

All data has been made available here and in the annexures.

## Supplementary Materials

The following supporting information can be downloaded at: https://www.mdpi.com/article/10.3390/nu16203564/s1, Table S1: The research question and detailed selection criteria; Table S2: Key words and the adjusted search terms as per searched electronic databases [updated on 13 August 2024]; Table S3: List of studies excluded after full-text assessment and reasons for exclusion; Table S4: Study characteristics of the included studies; Table S5: Meta regression using covariates; Table S6. Grade of evidence# in complete case analysis and after outlier removal; Figure S1: Forest plot showing subgroup analysis based on number of strains; Figure S2: Forest plot showing subgroup analysis based on treatment duration; Figure S3: Forest plot showing subgroup analysis based on study population; Figure S4: Forest plot showing subgroup analysis based on use of concomitant medications; Figure S5 Forest plot showing subgroup analysis based on region; Figure S6: Forest plot showing subgroup analysis based on RoB; Figure S7: Forest plot showing leave-one-out analysis; Figure S8: Forest plot showing pooled estimates after outliers’ removal; Figure S9: Risk of bias plot–domain wise; Figure S10: Funnel plot after outlier removal.
