# Supplementary material for: Effect of Probiotics Supplementation on Cortisol Levels: A Systematic Review and Meta-Analysis
Source: Nutrients. 2024 Oct 21;16(20):3564. doi: 10.3390/nu16203564 (PMC11510182; doi:10.3390/nu16203564)

Table S1: The research question and detailed selection criteria

**Research question – Does probiotics administration decrease cortisol levels in the participants?**<br>Null Hypothesis (H0): Probiotics do not decrease cortisol levels<br>Alternative Hypothesis (H1): Probiotics decrease cortisol levels		
Inclusion		Exclusion
Participants	Participants of all age groups.<br>No specific medical condition or disorder requirement.	Animals, in vitro, or other non-human studies
Intervention	Studies involving the administration of probiotics<br>Probiotic interventions can be standalone or part of a combination therapy	Studies using prebiotics, synbiotics, or postbiotics without a specific focus on probiotics.<br>Studies where probiotics are part of a complex treatment regimen making it challenging to isolate probiotic effects.
Comparison	Studies with a control group that did not receive probiotics (placebo or standard treatment).	Exclude studies lacking a clear control or comparison group.<br>Yogurt or other fermented foods in which specific probiotic organisms were not identified.
Outcome	Salivary/plasma/serum/urinary/fecal cortisol	Studies not assessing the effects of probiotics
Time point	For the studies evaluating stress, the samples analyzed just before or after time point maximum stress are included. Also, the sample within or at 30 min after waking up in case of morning sample, or if only one sample is taken, or Cmax	Measurement taken during the follow-up period
Study Designs	RCT<br>Geography-Global level<br>Date of Search- Published till<br>Language: English	Exclude non-randomized studies, observational studies, case reports, reviews, and other non-RCT designs.<br>Language: Non-English

Table S2: Key words and the adjusted search terms as per searched electronic databases [updated on 08/13/2024]

**List of key concepts, synonyms, and acronyms**			
Probiotics, psychobiotics, prebiotics, synbiotics, lactobacillus, bifidobacterium, saccharomyces, streptococcus, enterococcus, Propionibacterium, bacillus, lacticaseibacillus			
**Database**	**No.**	**Search Query**	**Results**
Pubmed	#1	prebiotics[MeSH Terms] OR "prebiotic*"[Title/Abstract] OR "synbiotics"[MeSH Terms] OR "synbiotic*"[Title/Abstract] OR "probiotics"[MeSH Terms] OR "probiotic*"[Title/Abstract] OR "bifidobacterium"[MeSH Terms] OR "bifidobacter*"[Title/Abstract] OR "saccharomyces"[MeSH Terms] OR "saccharomyc*"[Title/Abstract] OR "streptococcus"[MeSH Terms] OR "streptococc*"[Title/Abstract] OR "enterococcus"[MeSH Terms] OR "enterococc*"[Title/Abstract] OR "propionibacterium"[MeSH Terms] OR "propionibacteri*"[Title/Abstract] OR "Lacticaseibacillus"[MeSH Terms] OR "lacticaseibacill*"[Title/Abstract] OR "bacillus"[MeSH Terms] OR "bacill*"[Title/Abstract] OR "lactobacillus"[MeSH Terms] OR "lactobacill*"[Title/Abstract] OR "psychobiotic*"[Title/Abstract]	555,734
	#2	hydrocortisone[MeSH Terms] OR "cortisol"[Title/Abstract]	107,394
	#3	randomized controlled trial[Publication Type] OR "controlled clinical trial"[Publication Type] OR "randomized"[Title/Abstract] OR "randomised"[Title/Abstract] OR "placebo"[Title/Abstract] OR "drug therapy"[MeSH Subheading] OR "randomly"[Title/Abstract] OR "trial"[Title/Abstract] OR "groups"[Title/Abstract]	6,143,817
	#4	#1 AND #2 AND #3	251
	#5	#1 AND #2 AND #3 Filters: Humans	94
	#6	#1 AND #2 AND #3 Filters: Humans, English	88
	#7	("meta-analysis"[Publication Type] OR "systematic review"[Publication Type] OR "review"[Publication Type]) Filter:English, Humans	6,670
	#8	#6 NOT #7	88

EMBASE	#1	'prebiotic agent'/exp OR 'prebiotic*':ti,ab,kw OR 'synbiotic agent'/exp OR 'synbiotic*':ti,ab,kw OR 'probiotic agent'/exp OR 'probiotic*':ti,ab,kw OR 'Bifidobacterium'/exp OR 'bifidobacter*':ti,ab,kw OR 'Saccharomyces'/exp OR 'saccharomyc*':ti,ab,kw OR 'Streptococcus'/exp OR 'streptococc*':ti,ab,kw OR 'Enterococcus'/exp OR 'enterococc*':ti,ab,kw OR 'Propionibacterium'/exp OR 'propionibacteri*':ti,ab,kw OR 'Lacticaseibacillus'/exp OR 'lacticaseibacill*':ti,ab,kw OR 'Bacillus'/exp OR 'bacill*':ti,ab,kw OR 'Lactobacillus'/exp OR 'lactobacill*':ti,ab,kw OR 'psychobiotic*':ti,ab,kw	719,176
	#2	'hydrocortisone'/exp OR 'cortisol':ti,ab,kw	195,047
	#3	'randomized controlled trial':it OR 'controlled clinical trial':it OR 'randomized':ti,ab,kw OR 'randomised':ti,ab,kw OR 'placebo':ti,ab,kw OR 'drug therapy' OR 'randomly':ti,ab,kw OR 'trial':ti,ab,kw OR 'groups':ti,ab,kw	10,292,486
	#4	#1 AND #2 AND #3	1,321
	#5	#1 AND #2 AND #3 Filters: Humans	1,063
	#6	#1 AND #2 AND #3 Filters: Humans, English	1,001
	#7	('meta-analysis':it OR 'systematic review':it OR 'review':it)	3,289,298
	#8	#7 NOT #8	739
Scopus	#1	INDEXTERMS(prebiotics) OR TITLE-ABS(prebiotic*) OR INDEXTERMS(synbiotics) OR TITLE-ABS(synbiotic*) OR INDEXTERMS(probiotics) OR TITLE-ABS(probiotic*) OR INDEXTERMS(bifidobacterium) OR TITLE-ABS(bifidobacter*) OR INDEXTERMS(saccharomyces) OR TITLE-ABS(saccharomyc*) OR INDEXTERMS(streptococcus) OR TITLE-ABS(streptococc*) OR INDEXTERMS(enterococcus) OR TITLE-ABS(enterococc*) OR INDEXTERMS(propionibacterium) OR TITLE-ABS(propionibacteri*) OR INDEXTERMS(Lacticaseibacillus) OR TITLE-ABS(lacticaseibacill*) OR INDEXTERMS(bacillus) OR TITLE-ABS(bacill*) OR INDEXTERMS(lactobacillus) OR TITLE-ABS(lactobacill*) OR TITLE-ABS(psychobiotic*)	862,989
	#2	INDEXTERMS(hydrocortisone) OR TITLE-ABS(cortisol)	182,104
	#3	DOCTYPE ("Clinical Trial") OR INDEXTERMS ("Clinical Trials as Topic") OR TITLE-ABS ("randomised clinical trial") OR TITLE-ABS ("randomized clinical trial") OR TITLE-ABS (randomi*) OR TITLE-ABS ("randomised trial") OR TITLE-ABS ("randomized trial") OR TITLE-ABS ("Clinical Trial") OR TITLE-ABS (intervention) OR TITLE-ABS ("control study")	3,315,551

	#4	#1 AND #2 AND #3	301
	#5	#1 AND #2 AND #3 Filters: Humans, English	248
	#6	TITLE(meta-analysis) OR TITLE("systematic review") OR TITLE(review)	1,435,705
	#7	#5 not #6	215
Web of Science	#1	ALL=prebiotics OR (TI=prebiotic* OR AB=prebiotic*) OR ALL=synbiotics OR (TI=synbiotic* OR AB=synbiotic*) OR ALL=probiotics OR (TI=probiotic* OR AB=probiotic*) OR ALL=bifidobacterium OR (TI=bifidobacter* OR AB=bifidobacter*) OR ALL=saccharomyces OR (TI=saccharomyc* OR AB=saccharomyc*) OR ALL=streptococcus OR (TI=streptococc* OR AB=streptococc*) OR ALL=enterococcus OR (TI=enterococc* OR AB=enterococc*) OR ALL=propionibacterium OR (TI=propionibacteri* OR AB=propionibacteri*) OR ALL=Lacticaseibacillus OR (TI=lacticaseibacill* OR AB=lacticaseibacill*) OR ALL=bacillus OR (TI=bacill* OR AB=bacill*) OR ALL=lactobacillus OR (TI=lactobacill* OR AB=lactobacill*) OR (TI=psychobiotic* OR AB=psychobiotic*)	683,338
	#2	ALL=hydrocortisone OR (TI=cortisol OR AB=cortisol)	96,685
	#3	ALL="randomized controlled trial" OR ALL="controlled clinical trial" OR (TI=randomized OR AB=randomized) OR (TI=randomised OR AB=randomised) OR (TI=placebo OR AB=placebo) OR ALL="drug therapy" OR (TI=randomly OR AB=randomly) OR (TI=trial OR AB=trial) OR (TI=groups OR AB=groups)	**7,932,475**
	#4	#1 AND #2 AND #3	342
	#5	#1 AND #2 AND #3 Filters:English	338
	#6	(TI=meta-analysis OR TI="systematic review" OR TI=review)	1,169,298
	#7	#5 NOT #6	331
CINAHL	#1	(MH prebiotics+) OR (TI prebiotic* OR AB prebiotic*) OR (MH synbiotics+) OR (TI synbiotic* OR AB synbiotic*) OR (MH probiotics+) OR (TI probiotic* OR AB probiotic*) OR (MH bifidobacterium+) OR (TI bifidobacter* OR AB bifidobacter*) OR (MH saccharomyces+) OR (TI saccharomyc* OR AB saccharomyc*) OR (MH streptococcus+) OR (TI streptococc* OR AB streptococc*) OR (MH enterococcus+) OR (TI enterococc* OR AB enterococc*) OR (MH propionibacterium+) OR (TI propionibacteri* OR AB	39,856

		propionibacteri*) OR (MH Lacticaseibacillus+) OR (TI lacticaseibacill* OR AB lacticaseibacill*) OR (MH bacillus+) OR (TI bacill* OR AB bacill*) OR (MH lactobacillus+) OR (TI lactobacill* OR AB lactobacill*) OR (TI psychobiotic* OR AB psychobiotic*)	
	#2	(MH hydrocortisone+) OR (TI cortisol OR AB cortisol)	13,470
	#3	((MH randomized controlled trials OR MH double-blind studies OR MH single-blind studies OR MH random assignment OR MH pretest-posttest design OR MH cluster sample OR TI (randomised OR randomized) OR AB (random*) OR TI (trial) OR (MH (sample size) AND AB (assigned OR allocated OR control)) OR MH (placebos) OR PT (randomized controlled trial) OR AB (control W5 group) OR MH (crossover design) OR MH (comparative studies) OR AB (cluster W3 RCT)) NOT ((MH animals+ OR MH animal studies OR TI (animal model*)) NOT MH human)) OR (TX allocat* random* OR (MH "Quantitative Studies") OR (MH "Placebos") OR TX placebo* OR TX random* allocat* OR (MH "Random Assignment") OR TX randomi* control* trial* OR TX ((singl* N1 blind*) or (singl* N1 mask*)) OR TX ((doubl* N1 blind*) or (doubl* N1 mask*)) OR TX ((tripl* N1 blind*) or (tripl* N1 mask*)) OR TX ((trebl* N1 blind*) or (trebl* N1 mask*)) OR (TX clinic* N1 trial*) OR PT Clinical trial OR (MH "Clinical Trials+"))	2,473,129
	#4	#1 AND #2 AND #3	32
	#5	#1 AND #2 AND #3 Filter:English	32
Cochrane Library	#1	[mh prebiotics] OR prebiotic*:ti,ab OR [mh synbiotics] OR synbiotic*:ti,ab OR [mh probiotics] OR probiotic*:ti,ab OR [mh bifidobacterium] OR bifidobacter*:ti,ab OR [mh saccharomyces] OR saccharomyc*:ti,ab OR [mh streptococcus] OR streptococc*:ti,ab OR [mh enterococcus] OR enterococc*:ti,ab OR [mh propionibacterium] OR propionibacteri*:ti,ab OR [mh Lacticaseibacillus] OR lacticaseibacill*:ti,ab OR [mh bacillus] OR bacill*:ti,ab OR [mh lactobacillus] OR lactobacill*:ti,ab OR psychobiotic*:ti,ab	23,686
	#2	[mh hydrocortisone] OR cortisol:ti,ab	17,453
	#3	#1 AND #2	166
	#4	#1 AND #2 Filters:Only trials	166

	#5	#1 AND #2 Filters:Only trials, English	162
Proquest	#1	TI,AB(prebiotic*) OR TI,AB(synbiotic*) OR TI,AB(probiotic*) OR TI,AB(bifidobacter*) OR TI,AB(saccharomyc*) OR TI,AB(streptococc*) OR TI,AB(enterococc*) OR TI,AB(propionibacteri*) OR TI,AB(lacticaseibacill*) OR TI,AB(bacill*) OR TI,AB(lactobacill*) OR TI,AB(psychobiotic*)	220,407
	#2	TI,AB(cortisol) OR TI,AB(hydrocortisone)	32,507
	#3	NOFT("randomized controlled trial") OR NOFT("controlled clinical trial") OR TI,AB(randomized) OR TI,AB(randomised) OR TI,AB(placebo) OR NOFT("drug therapy") OR TI,AB(randomly) OR TI,AB(trial) OR TI,AB(groups)	16,242,149
	#4	#1 AND #2 AND #3	173
	#5	#1 AND #2 AND #3 Filter:English	172
	#6	#5 Available literature	171
WoS Preprint Citation Index	#1	(TI=prebiotic* OR AB=prebiotic*) OR (TI=synbiotic* OR AB=synbiotic*) OR (TI=probiotic* OR AB=probiotic*) OR (TI=bifidobacter* OR AB=bifidobacter*) OR (TI=saccharomyc* OR AB=saccharomyc*) OR (TI=streptococc* OR AB=streptococc*) OR (TI=entero cocc* OR AB=enterococc*) OR (TI=propionibacteri* OR AB=propionibacteri*) OR (TI=lacticaseibacill* OR AB=lac ticaseibacill*) OR (TI=bacill* OR AB=bacill*) OR (TI=lactobacill* OR AB=lactobacill*) OR (TI=psychobiotic * OR AB=psychobiotic*)	5,057
	#2	(TI=cortisol OR AB=cortisol) OR (TI = hydrocortisone OR AB = hydrocortisone)	137
	#3	(TI=randomized OR AB=randomized) OR (TI=randomised OR AB=randomised) OR (TI=placebo OR AB=placebo) OR (TI=randomly OR AB=randomly) OR (TI=trial OR AB=trial) OR (TI=groups OR AB=groups)	223,095
	#4	#1 AND #2 AND #3	2

Table S3: List of studies excluded after full-text assessment and reasons for exclusion

Title	Study	Identifier	Notes
Probiotic Mixture Containing Lactobacillus helveticus , Bifidobacterium longum and Lactiplantibacillus plantarum Affects Brain Responses to an Arithmetic Stress Task in Healthy Subjects: A Randomised Clinical Trial and Proof-of-Concept Study.	EdebolCarlman 2022	https://www.mdpi.com/2072-6643/14/7/1329	Wrong intervention
Influence of mechanical debridement with adjunct probiotic therapy on clinical status and salivary cortisol levels in patients with periodontal inflammation.	Alhamoudi 2023	https://www.europeanreview.org/article/33758	Wrong study design/non-randomized
Multi-Strain Probiotic Mixture Affects Brain Morphology and Resting State Brain Function in Healthy Subjects: An RCT.	Rode 2022	https://www.mdpi.com/2073-4409/11/18/2922	Wrong intervention
Lactobacillus rhamnosus CNCM I-3690 decreases subjective academic stress in healthy adults: a randomized placebo-controlled trial.	Wauters 2022	https://onlinelibrary.wiley.com/doi/10.1111/nmo.13671	data not available
The Effect of a Probiotic Complex on the Gut-Brain Axis: A Translational Study.	Nobile 2022	https://karger.com/nps/article-abstract/81/2/116/828847/The-Effect-of-a-Probiotic-Complex-on-the-Gut-Brain?redirectedFrom=fulltext	Wrong intervention
The alteration of stress-related physiological parameters after probiotics administration in oral surgeons with different degrees of surgical experience.	Pacifici 2020	https://www.clinicaterapeutica.it/2020/171/3/05_BALLINI-INCHINGOLO.pdf	data not available

Bifidobacterium longum 1714â„¢ Strain Modulates Brain Activity of Healthy Volunteers During Social Stress.	Wang 2019	https://journals.lww.com/ajg/fulltext/2019/07000/bifidobacterium_longum_1714__strain_modulates.25.aspx	Wrong outcomes
Effects Of A Probiotic Formulation After Experimentally Induced Stress In An Adult Population	QuesadaRodriguez 2023	https://linkinghub.elsevier.com/retrieve/pii/S2405457722010622	Abstract/Posters
Feed your microbes to deal with stress: a psychobiotic diet impacts microbial stability and perceived stress in a healthy adult population	Berding 2023	https://www.nature.com/articles/s41380-022-01817-y	Wrong intervention
Interactions between symptoms and psychological status in irritable bowel syndrome: An exploratory study of the impact of a probiotic combination	Groeger 2023	https://onlinelibrary.wiley.com/doi/10.1111/nmo.14477	Wrong study design/non-randomized
Effects of the multidomain intervention with nutritional supplements on cognition and gut microbiome in early symptomatic Alzheimerâ€™s disease: a randomized controlled trial	Lee 2023	https://www.frontiersin.org/journals/aging-neuroscience/articles/10.3389/fnagi.2023.1266955/full	Wrong intervention
Efficacy of a food supplement based on S-adenosyl methionine and probiotic strains in subjects with subthreshold depression and mild-to-moderate depression: A monocentric, randomized, cross-over, double-blind, placebo-controlled clinical trial	Ullah 2022	https://www.sciencedirect.com/science/article/pii/S0753332222013191?via%3Dihub	Wrong intervention
Microbiome Modulators and Mood Disorders: Using a Multi-Strain Probiotic - Bio-KultÂ® Advanced - in Patients With Low Mood	Aswani 2022	https://www.cambridge.org/core/journals/bjpsych-open/article/microbiome-modulators-and-mood-disorders-using-a-multistrain-probiotic-biokult-advanced-in-patients-with-low-mood/FC5978D81CABCD5E5D088441F248F9DF	Abstract/Posters

Routine Administration of a Multispecies Probiotic Containing Bifidobacterium and Lactobacillus to Very Low Birth Weight Infants Had No Significant Impact on the Incidence of Necrotizing Enterocolitis	Juber 2021	https://www.frontiersin.org/journals/pediatrics/articles/10.3389/fped.2021.757299/full	Wrong study design/non-randomized
Probiotics-induced changes in gut microbial composition and its effects on cognitive performance after stress: exploratory analyses	Bloemendaal 2021	https://www.nature.com/articles/s41398-021-01404-9	Wrong study design/non-randomized
Efficacy of a multispecies probiotic as adjunctive therapy in generalized anxiety disorder: a double blind, randomized, placebo-controlled trial	Eskandarzadeh 2021	https://www.tandfonline.com/doi/full/10.1080/1028415X.2019.1598669	data not available
LB946 Improvements in acne-prone skin quality correlate with a reduction in saliva cortisol levels after use of an 8-week 3-step topical regimen	Skobowiat 2020	https://linkinghub.elsevier.com/retrieve/pii/S0022202X20315335	Abstract/Posters
Effect of lactobacillus rhamnosus strain on stress-related intestinal permeability in healthy adults (ProSPer): A randomized, doubleblind placebo-controlled trial	Wauters 2019	https://onlinelibrary.wiley.com/doi/10.1111/nmo.13671	Abstract/Posters
Differences in psychosocial behavior, salivary IgA, and oral microbiota in student-athletes and sedentary college students	Costan 2018	https://journals.aai.org/jimmunol/article/200/1_Supplement/118.17/59913/Differences-in-psychosocial-behavior-salivary-IgA	Abstract/Posters
Effect of coordinated probiotic/prebiotic/phytobiotic supplementation on microbiome balance and psychological mood state in healthy stressed adults	Talbott 2018	https://faseb.onlinelibrary.wiley.com/doi/abs/10.1096/fasebj.2018.32.1_supplement.lb122	Full text unavailable

Bifidobacterium longum 1714 reduces stress-induced changes in anxiety and salivary cortisol and alters neurocognitive performance and resting EEG in healthy volunteers	Allen 2016	https://journals.lww.com/psychosomaticmedicine/citation/2016/04000/abstracts_of_the_74th_annual_meeting_of_the.16.aspx	Abstract/Posters
Bifidobacterium bifidum R0071 decreases stress-associated diarrhoea-related symptoms and self-reported stress: A secondary analysis of a randomised trial	Culpepper 2016	https://brill.com/view/journals/bm/7/3/article-p327_3.xml	data not available
Randomized, placebo-controlled trial of Lactobacillus rhamnosus GG as treatment of atopic dermatitis in infancy	Gruber 2007	https://onlinelibrary.wiley.com/doi/10.1111/j.1398-9995.2007.01543.x	Wrong outcomes
Influence of continuous ingestion of lactobacillus pentosus strain B240 on saliva components and properties in healthy adults- A randomized, double-blind, placebo-controlled study	Shimizu 2018	https://jglobal.jst.go.jp/en/detail?JGLOBAL_ID=201802266753123951	Full text unavailable
Intake of MPRO3 over 4 Weeks Reduces Glucose Levels and Improves Gastrointestinal Health and Metabolism	Lee 2022	https://www.mdpi.com/2076-2607/10/1/88	Wrong intervention
Psychobiotic Supplementation of PS128â„¢ Improves Stress, Anxiety, and Insomnia in Highly Stressed Information Technology Specialists: A Pilot Study	Wu 2021	https://www.frontiersin.org/journals/nutrition/articles/10.3389/fnut.2021.614105/full	Wrong study design/non-randomized
Effect of Probiotic Therapy on Neuropsychiatric Manifestations in Children with Multiple Neurotransmitter Disorders: A Study	Matis 2023	https://www.mdpi.com/2227-9059/11/10/2643	Wrong study design/non-randomized

Is the gut microbiota bacterial abundance and composition associated with intestinal epithelial injury, systemic inflammatory profile, and C gastrointestinal symptoms in response to exertional-heat stress?	Bennett 2020	https://linkinghub.elsevier.com/retrieve/pii/S1440244020306629	Wrong study design/non-randomized
Gut microbiome patterns depending on children's psychosocial stress: Reports versus biomarkers	Michels 2019	https://www.sciencedirect.com/science/article/abs/pii/S0889159118312145?via%3Dihub	Wrong study design/non-randomized
The effectiveness of a Lactobacillus probiotic on measures of psychosocial health in adults diagnosed with subthreshold depression: a double-blind, randomised, placebo-controlled trial	Moschonis 2024	https://www.cambridge.org/core/journals/proceedings-of-the-nutrition-society/article/effectiveness-of-a-lactobacillus-probiotic-on-measures-of-psychosocial-health-in-adults-diagnosed-with-subthreshold-depression-a-doubleblind-randomised-placebocontrolled-trial/AD884D1A0315BA9536FF967A7884415B	Full text unavailable
A Preliminary Study on Effect of Lactobacillus paracasei HII01 on Cortisol in Fatigue Subjects	Lalitsuradej 2019	https://ieeexplore.ieee.org/document/8955402/	Wrong study design/non-randomized
Psychobiotic Effects on Anxiety Are Modulated by Lifestyle Behaviors: A Randomized Placebo-Controlled Trial on Healthy Adults.	Morales-Torres 2023	https://www.mdpi.com/2072-6643/15/7/1706	Wrong outcomes
A randomized trial of probiotic supplementation in nurses to reduce stress and viral illness.	Slykerman 2022	https://www.nature.com/articles/s41598-022-19104-9	Wrong outcomes

The efficacy of probiotics and/or n-3 long-chain polyunsaturated fatty acids intervention on maternal prenatal and postnatal depressive and anxiety symptoms among overweight and obese women.	Hulkkonen 2021	https://www.sciencedirect.com/science/article/pii/S016503272100330X?via%3Dihub	Wrong outcomes
Metabolic Status Influences Probiotic Efficacy for Depression-PRO-DEMET Randomized Clinical Trial Results.	Gawlik-Kotelnicka 2024	https://www.mdpi.com/2072-6643/16/9/1389	Wrong outcomes

Table S4: Study characteristics of the included studies

Separate excel file attached with the supplementary

Table S5: Meta regression using covariates

Mixed-Effects Model (k = 44; tau^2 estimator: ML)							
tau^2 (estimated amount of residual heterogeneity):	1.2497	(SE = 0.28					
tau (square root of estimated tau^2 value):	1.1179						
I^2 (residual heterogeneity / unaccounted variability):	95.36%						
H^2 (unaccounted variability / sampling variability):	21.55						
R^2 (amount of heterogeneity accounted for):	18.42%						
Test for Residual Heterogeneity: QE(df = 39) = 571.1808, p-val <.0001							
Test of Moderators (coefficients 2:5): F(df1 = 4, df2 = 39) = 2.0412, p-val = 0.1075							
Model Results:							
	estimate	se	tval	df	pval	ci.lb	ci.ub
intrcpt	0.061	0.5199	0.1174	39	0.9072	-0.9906	1.1127
trt_dur_wk	0.0604	0.0401	1.5049	39	0.1404	-0.0208	0.1415
Strains	-0.0532	0.0638	-0.8335	39	0.4096	-0.1823	0.0759
Overall_Sex_M	-0.0216	0.0086	-2.5152	39	0.0161	-0.0389	-0.0042*
Overall_Sex_F	-0.0023	0.0054	-0.4202	39	0.6766	-0.0131	0.0086

Signif. codes:	0 ‘***’	0.001 ‘	**’ 0.01	‘*’	0.05 ‘.’	0.1 ‘ ’ 1	

Table S6. Grade of evidence# in complete case analysis and after outlier removal

Certainty assessment							№ of patients		Effect		Certainty	Importance
№ of studies	Study design	Risk of bias	Inconsistency	Indirectness	Imprecision	Other considerations	Probiotics	Control	Relative (95% CI)	Absolute (95% CI)		
**Cortisol - Complete case analysis**												
46	Randomised trials	Serious	Serious	Not serious	Not serious	None	1771	1745	-	SMD - **0.45** (-0.83 to -0.07)	⊕⊕◯◯ Low	High heterogeneity and risk of bias
**Cortisol - after outlier removal**												
38	Randomised trials	Serious	Not serious	Not serious	Not serious	None	1385	1357	-	SMD - **0.18** (-0.28 to -0.08)	⊕⊕⊕◯ Moderate	

CI: confidence interval; **SMD:** standardised mean difference; #GRADEpro GDT: GRADEpro Guideline Development Tool [Software]. McMaster University and Evidence Prime, 2024

Figure S1: Forest plot showing subgroup analysis based on number of strains

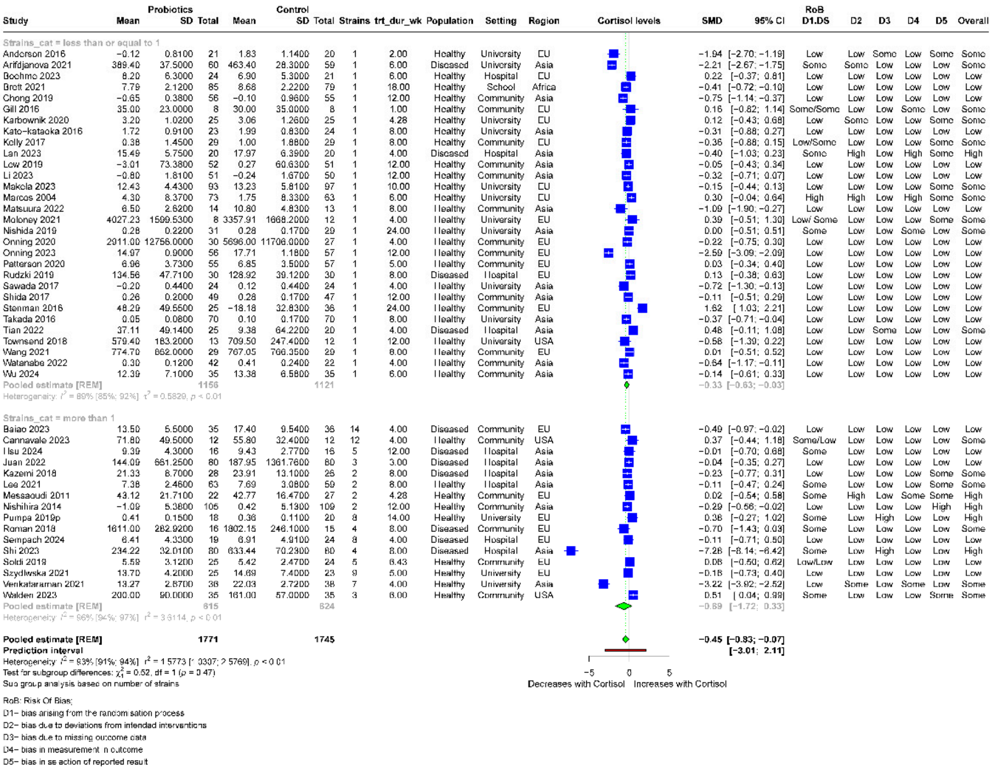

Figure S2: Forest plot showing subgroup analysis based on treatment duration

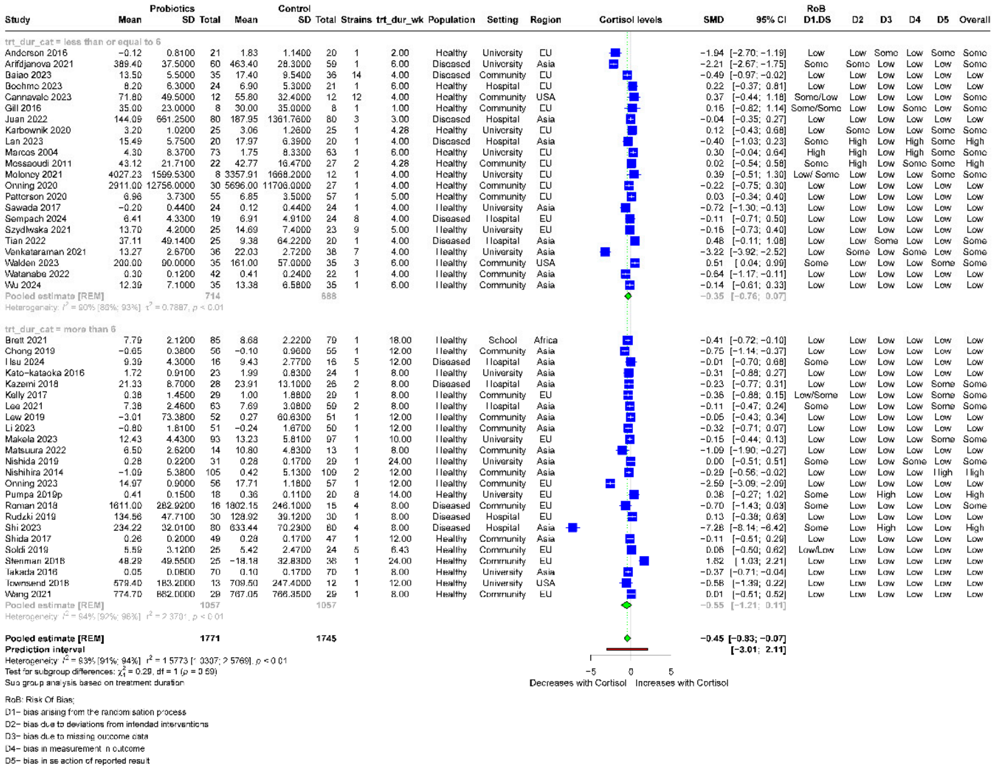

Figure S3: Forest plot showing subgroup analysis based on study population

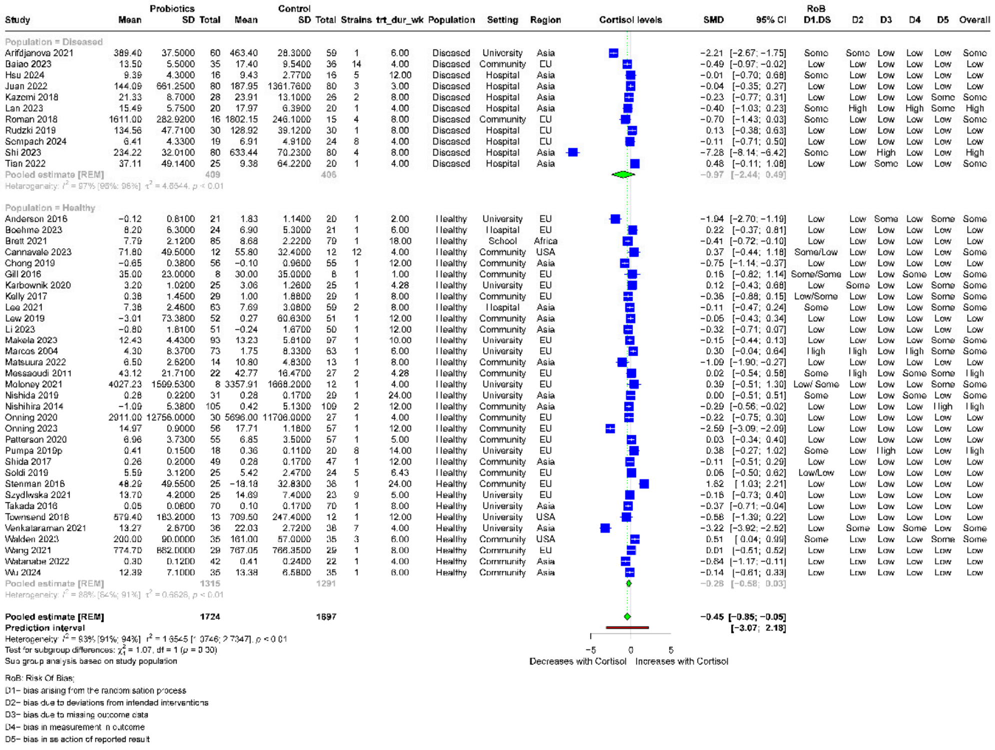

Figure S4: Forest plot showing subgroup analysis based on use of concomitant medications

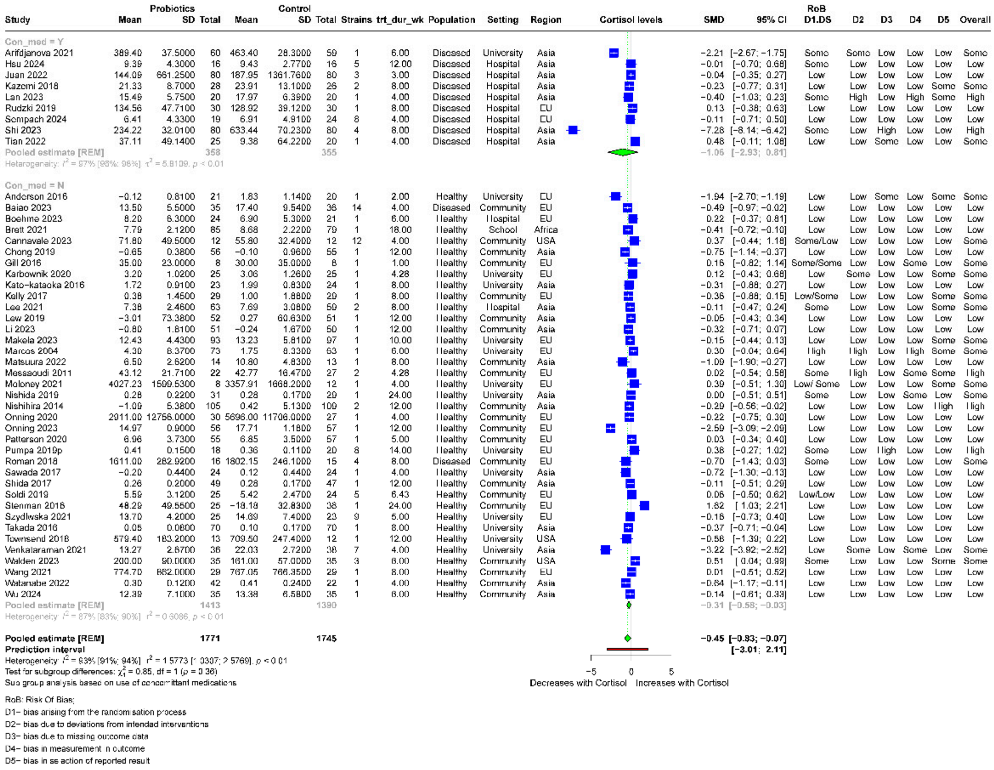

Figure S5 Forest plot showing subgroup analysis based on region

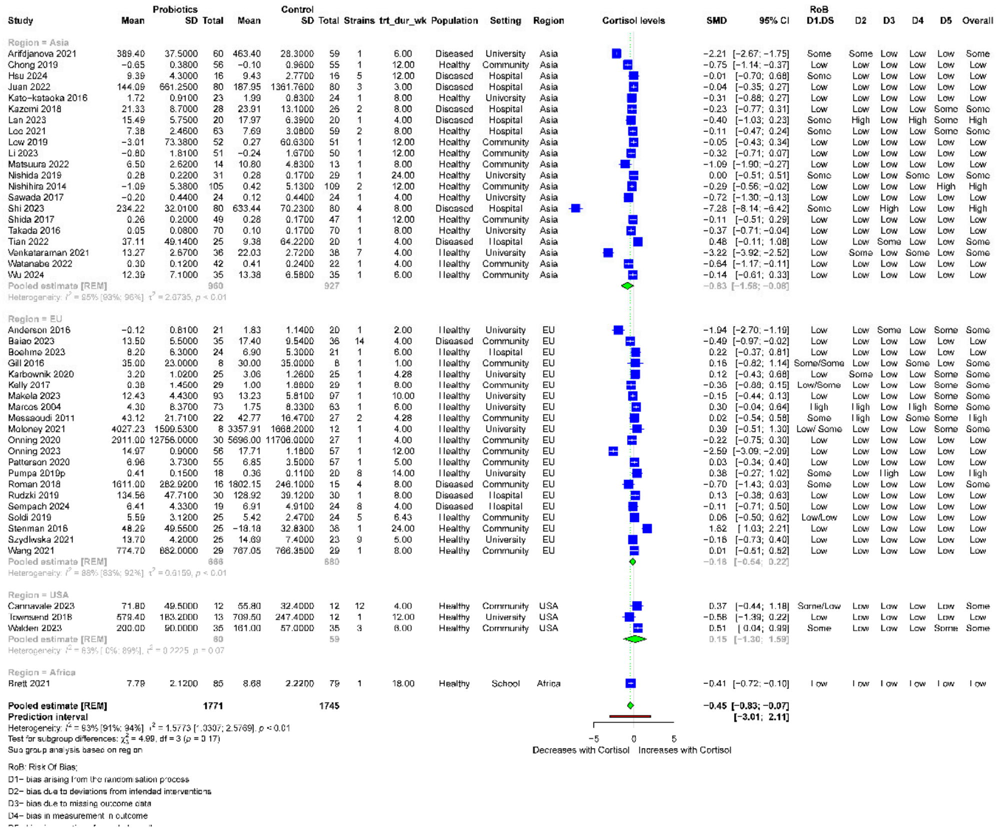

Figure S6: Forest plot showing subgroup analysis based on RoB

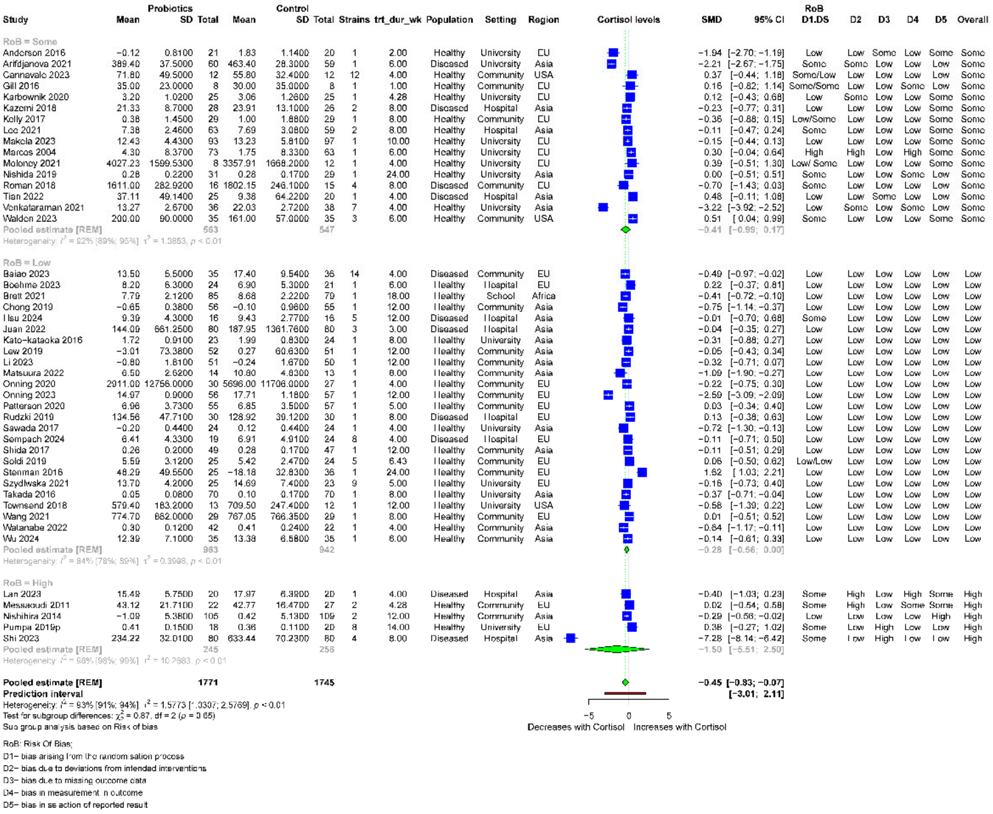

Figure S7: Forest plot showing leave-one-out analysis

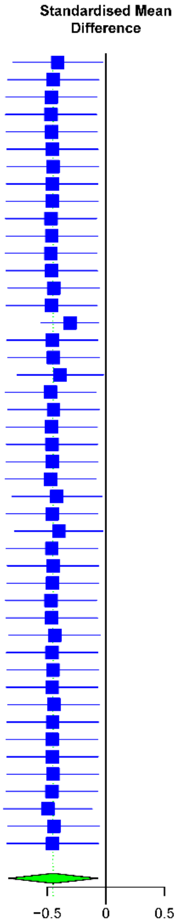

Study	SMD	95%−CI	P−value	Tau2	Tau	I2
Omitting Arifdjanova 2021	−0.41	[−0.79; −0.03]	0.04	1.5408	1.2413	92%
Omitting Baiao 2023	−0.45	[−0.84; −0.06]	0.03	1.6160	1.2712	93%
Omitting Boehme 2023	−0.47	[−0.85; −0.08]	0.02	1.6054	1.2670	93%
Omitting Cannavale 2023	−0.47	[−0.86; −0.08]	0.02	1.6006	1.2652	93%
Omitting Karbownik 2020	−0.46	[−0.85; −0.07]	0.02	1.6082	1.2682	93%
Omitting Kazemi 2018	−0.46	[−0.85; −0.06]	0.02	1.6149	1.2708	93%
Omitting Lan 2023	−0.45	[−0.84; −0.06]	0.02	1.6160	1.2712	93%
Omitting Lee 2021	−0.46	[−0.85; −0.07]	0.02	1.6132	1.2701	93%
Omitting Makela 2023	−0.46	[−0.85; −0.07]	0.02	1.6139	1.2704	93%
Omitting Marcos 2004	−0.47	[−0.86; −0.08]	0.02	1.6020	1.2657	93%
Omitting Messaoudi 2011	−0.46	[−0.85; −0.07]	0.02	1.6108	1.2692	93%
Omitting Pumpa 2019p	−0.47	[−0.86; −0.08]	0.02	1.5999	1.2649	93%
Omitting Patterson 2020	−0.46	[−0.85; −0.07]	0.02	1.6103	1.2690	93%
Omitting Roman 2018	−0.45	[−0.84; −0.05]	0.03	1.6146	1.2707	93%
Omitting Rudzki 2019	−0.46	[−0.85; −0.07]	0.02	1.6080	1.2680	93%
Omitting Shi 2023	−0.31	[−0.55; −0.06]	0.02	0.5929	0.7700	87%
Omitting Szydlwska 2021	−0.46	[−0.85; −0.07]	0.02	1.6141	1.2705	93%
Omitting Townsend 2018	−0.45	[−0.84; −0.06]	0.03	1.6157	1.2711	93%
Omitting Venkataraman 2021	−0.39	[−0.76; −0.02]	0.04	1.4374	1.1989	92%
Omitting Walden 2023	−0.47	[−0.86; −0.08]	0.02	1.5935	1.2624	93%
Omitting Watanabe 2022	−0.45	[−0.84; −0.06]	0.03	1.6152	1.2709	93%
Omitting Gill 2016	−0.46	[−0.85; −0.07]	0.02	1.6078	1.2680	93%
Omitting Hsu 2024	−0.46	[−0.85; −0.07]	0.02	1.6115	1.2694	93%
Omitting Wu 2024	−0.46	[−0.85; −0.07]	0.02	1.6137	1.2703	93%
Omitting Tian 2022	−0.47	[−0.86; −0.08]	0.02	1.5952	1.2630	93%
Omitting Anderson 2016	−0.42	[−0.80; −0.03]	0.03	1.5647	1.2509	92%
Omitting Sempach 2024	−0.46	[−0.85; −0.07]	0.02	1.6132	1.2701	93%
Omitting Onning 2023	−0.40	[−0.78; −0.02]	0.04	1.5054	1.2270	92%
Omitting Wang 2021	−0.46	[−0.85; −0.07]	0.02	1.6109	1.2692	93%
Omitting Brett 2021	−0.45	[−0.84; −0.06]	0.02	1.6160	1.2712	93%
Omitting Nishihira 2014	−0.45	[−0.85; −0.06]	0.02	1.6154	1.2710	93%
Omitting Moloney 2021	−0.47	[−0.86; −0.08]	0.02	1.6002	1.2650	93%
Omitting Soldi 2019	−0.46	[−0.85; −0.07]	0.02	1.6098	1.2688	93%
Omitting Matsuura 2022	−0.44	[−0.83; −0.05]	0.03	1.6069	1.2676	93%
Omitting Juan 2022	−0.46	[−0.85; −0.07]	0.02	1.6119	1.2696	93%
Omitting Takada 2016	−0.45	[−0.84; −0.06]	0.02	1.6159	1.2712	93%
Omitting Lew 2019	−0.46	[−0.85; −0.07]	0.02	1.6121	1.2697	93%
Omitting Chong 2019	−0.44	[−0.83; −0.05]	0.03	1.6139	1.2704	93%
Omitting Li 2023	−0.45	[−0.84; −0.06]	0.02	1.6156	1.2711	93%
Omitting Shida 2017	−0.46	[−0.85; −0.07]	0.02	1.6131	1.2701	93%
Omitting Onning 2020	−0.46	[−0.85; −0.06]	0.02	1.6148	1.2707	93%
Omitting Kelly 2017	−0.45	[−0.84; −0.06]	0.02	1.6159	1.2712	93%
Omitting Nishida 2019	−0.46	[−0.85; −0.07]	0.02	1.6111	1.2693	93%
Omitting Stenman 2016	−0.50	[−0.87; −0.12]	0.01	1.5136	1.2303	92%
Omitting Sawada 2017	−0.44	[−0.84; −0.05]	0.03	1.6144	1.2706	93%
Omitting Kato−kataoka 2016	−0.45	[−0.84; −0.06]	0.02	1.6155	1.2710	93%
**Random effects model**	**−0.45**	**[−0.83; −0.07]**	**0.02**	**1.5773**	**1.2559**	**93%**

Sensitivity Analysis
Leave−one−out Meta−Analysis

Figure S8: Forest plot showing pooled estimates after outliers' removal

Study	Probiotics Mean	Probiotics SD	Probiotics Total	Control Mean	Control SD	Control Total	Strains	trt_dur_wk	Population	Setting	Region	SMD	95% CI	RoB D1.DS	D2	D3	D4	D5	Overall
Anderson 2016	−0.12	0.8100	21	1.83	1.1400	20	1	2.00	Healthy	University	EU	−1.94	[−2.70; −1.19]	Low	Low	Some	Low	Some	Some
Arifdjanova 2021	389.40	37.5000	60	463.40	28.3000	59	1	6.00	Diseased	University	Asia	−2.21	[−2.67; −1.75]	Some	Some	Low	Low	Low	Some
Baiao 2023	13.50	5.5000	35	17.40	9.5400	36	14	4.00	Diseased	Community	EU	−0.49	[−0.97; −0.02]	Low	Low	Low	Low	Low	Low
Boehme 2023	8.20	6.3000	24	6.90	5.3000	21	1	6.00	Healthy	Hospital	EU	0.22	[−0.37; 0.81]	Low	Low	Low	Low	Low	Low
Brett 2021	7.79	2.1200	85	8.68	2.2200	79	1	18.00	Healthy	School	Africa	−0.41	[−0.72; −0.10]	Low	Low	Low	Low	Low	Low
Cannavale 2023	71.80	49.5000	12	55.80	32.4000	12	12	4.00	Healthy	Community	USA	0.37	[−0.44; 1.18]	Some/Low	Low	Low	Low	Low	Some
Chong 2019	−0.65	0.3800	56	−0.10	0.9600	55	1	12.00	Healthy	Community	Asia	−0.75	[−1.14; −0.37]	Low	Low	Low	Low	Low	Low
Gill 2016	35.00	23.0000	8	30.00	35.0000	8	1	1.00	Healthy	Community	EU	0.16	[−0.82; 1.14]	Some/Some	Low	Low	Some	Low	Some
Hsu 2024	9.39	4.3000	16	9.43	2.7700	16	5	12.00	Diseased	Hospital	Asia	−0.01	[−0.70; 0.68]	Some	Low	Low	Low	Low	Low
Juan 2022	144.09	661.2500	80	187.95	1361.7600	80	3	3.00	Diseased	Hospital	Asia	−0.04	[−0.35; 0.27]	Low	Low	Low	Low	Low	Low
Karbownik 2020	3.20	1.0200	25	3.06	1.2600	25	1	4.28	Healthy	University	EU	0.12	[−0.43; 0.68]	Low	Some	Low	Low	Some	Some
Kato-kataoka 2016	1.72	0.9100	23	1.99	0.8300	24	1	8.00	Healthy	University	Asia	−0.31	[−0.88; 0.27]	Low	Low	Low	Low	Low	Low
Kazemi 2018	21.33	8.7000	28	23.91	13.1000	26	2	8.00	Diseased	Hospital	Asia	−0.23	[−0.77; 0.31]	Low	Low	Low	Low	Some	Some
Kelly 2017	0.38	1.4500	29	1.00	1.8800	29	1	8.00	Healthy	Community	EU	−0.36	[−0.88; 0.15]	Low/Some	Low	Low	Low	Some	Some
Lan 2023	15.49	5.7500	20	17.97	6.3900	20	1	4.00	Diseased	Hospital	Asia	−0.40	[−1.03; 0.23]	Some	High	Low	High	Some	High
Lee 2021	7.38	2.4600	63	7.69	3.0800	59	2	8.00	Healthy	Hospital	Asia	−0.11	[−0.47; 0.24]	Some	Low	Low	Low	Some	Some
Lew 2019	−3.01	73.3800	52	0.27	60.6300	51	1	12.00	Healthy	Community	Asia	−0.05	[−0.43; 0.34]	Low	Low	Low	Low	Low	Low
Li 2023	−0.80	1.8100	51	−0.24	1.6700	50	1	12.00	Healthy	Community	Asia	−0.32	[−0.71; 0.07]	Low	Low	Low	Low	Low	Low
Makela 2023	12.43	4.4300	93	13.23	5.8100	97	1	10.00	Healthy	University	EU	−0.15	[−0.44; 0.13]	Low	Low	Low	Low	Some	Some
Marcos 2004	4.30	8.3700	73	1.75	8.3300	63	1	6.00	Healthy	University	EU	0.30	[−0.04; 0.64]	High	High	Low	High	Some	Some
Matsuura 2022	6.50	2.6200	14	10.80	4.8300	13	1	8.00	Healthy	Community	Asia	−1.09	[−1.90; −0.27]	Low	Low	Low	Low	Low	Low
Messaoudi 2011	43.12	21.7100	22	42.77	16.4700	27	2	4.28	Healthy	Community	EU	0.02	[−0.54; 0.58]	Some	High	Low	Some	Some	High
Moloney 2021	4027.23	1599.5300	8	3357.91	1668.2000	12	1	4.00	Healthy	University	EU	0.39	[−0.51; 1.30]	Low/ Some	Low	Low	Low	Some	Some
Nishida 2019	0.28	0.2200	31	0.28	0.1700	29	1	24.00	Healthy	University	Asia	0.00	[−0.51; 0.51]	Some	Low	Low	Some	Low	Some
Nishihira 2014	−1.09	5.3800	105	0.42	5.1300	109	2	12.00	Healthy	Community	Asia	−0.29	[−0.56; −0.02]	Low	Low	Low	Low	High	High
Onning 2020	2911.00	12756.0000	30	5696.00	11706.0000	27	1	4.00	Healthy	Community	EU	−0.22	[−0.75; 0.30]	Low	Low	Low	Low	Low	Low
Onning 2023	14.97	0.9000	56	17.71	1.1800	57	1	12.00	Healthy	Community	EU	−2.59	[−3.09; −2.09]	Low	Low	Low	Low	Low	Low
Patterson 2020	6.96	3.7300	55	6.85	3.5000	57	1	5.00	Healthy	Community	EU	0.03	[−0.34; 0.40]	Low	Low	Low	Low	Low	Low
Pumpa 2019p	0.41	0.1500	18	0.36	0.1100	20	8	14.00	Healthy	University	EU	0.38	[−0.27; 1.02]	Some	Low	High	Low	Low	High
Roman 2018	1611.00	282.9200	16	1802.15	246.1000	15	4	8.00	Diseased	Community	EU	−0.70	[−1.43; 0.03]	Some	Low	Low	Low	Low	Some
Rudzki 2019	134.56	47.7100	30	128.92	39.1200	30	1	8.00	Diseased	Hospital	EU	0.13	[−0.38; 0.63]	Low	Low	Low	Low	Low	Low
Sawada 2017	−0.20	0.4400	24	0.12	0.4400	24	1	4.00	Healthy	University	Asia	−0.72	[−1.30; −0.13]	Low	Low	Low	Low	Low	Low
Sempach 2024	6.41	4.3300	19	6.91	4.9100	24	8	4.00	Diseased	Hospital	EU	−0.11	[−0.71; 0.50]	Low	Low	Low	Low	Low	Low
Shi 2023	234.22	32.0100	80	633.44	70.2300	80	4	8.00	Diseased	Hospital	Asia	−7.28	[−8.14; −6.42]	Some	Low	High	Low	Low	High
Shida 2017	0.26	0.2000	49	0.28	0.1700	47	1	12.00	Healthy	Community	Asia	−0.11	[−0.51; 0.29]	Low	Low	Low	Low	Low	Low
Soldi 2019	5.59	3.1200	25	5.42	2.4700	24	5	6.43	Healthy	Community	EU	0.06	[−0.50; 0.62]	Low/Low	Low	Low	Low	Low	Low
Stenman 2016	48.29	49.5500	25	−18.18	32.8300	36	1	24.00	Healthy	Community	EU	1.62	[1.03; 2.21]	Low	Low	Low	Low	Low	Low
Szydlwska 2021	13.70	4.2000	25	14.69	7.4000	23	9	5.00	Healthy	University	EU	−0.16	[−0.73; 0.40]	Low	Low	Low	Low	Low	Low
Takada 2016	0.05	0.0600	70	0.10	0.1700	70	1	8.00	Healthy	University	Asia	−0.37	[−0.71; −0.04]	Low	Low	Low	Low	Low	Low
Tian 2022	37.11	49.1400	25	9.38	64.2200	20	1	4.00	Diseased	Hospital	Asia	0.48	[−0.11; 1.08]	Low	Low	Some	Low	Low	Some
Townsend 2018	579.40	183.2000	13	709.50	247.4000	12	1	12.00	Healthy	University	USA	−0.58	[−1.39; 0.22]	Low	Low	Low	Low	Low	Low
Venkataraman 2021	13.27	2.6700	36	22.03	2.7200	38	7	4.00	Healthy	University	Asia	−3.22	[−3.92; −2.52]	Low	Some	Low	Some	Low	Some
Walden 2023	200.00	90.0000	35	161.00	57.0000	35	3	6.00	Healthy	Community	USA	0.51	[0.04; 0.99]	Some	Low	Low	Low	Some	Some
Wang 2021	774.70	882.0000	29	767.05	766.3500	29	1	8.00	Healthy	Community	EU	0.01	[−0.51; 0.52]	Low	Low	Low	Low	Low	Low
Watanabe 2022	0.30	0.1200	42	0.41	0.2400	22	1	4.00	Healthy	Community	Asia	−0.64	[−1.17; −0.11]	Low	Low	Low	Low	Low	Low
Wu 2024	12.39	7.1000	35	13.38	6.5800	35	1	6.00	Healthy	Community	Asia	−0.14	[−0.61; 0.33]	Low	Low	Low	Low	Low	Low
**Pooled estimate [REM]**			**1771**			**1745**						**−0.18**	**[−0.28; −0.08]**						
**Prediction interval**													**[−0.71; 0.35]**						

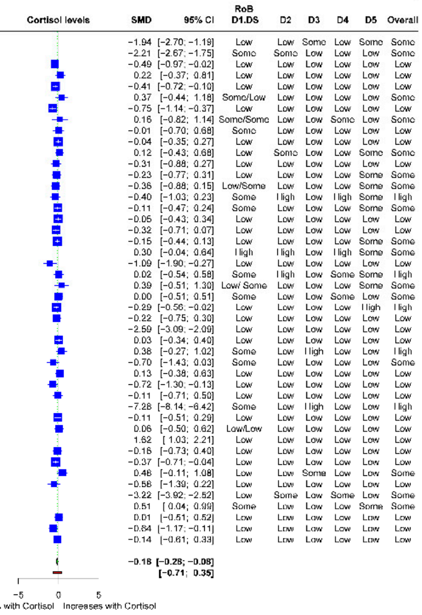

Heterogeneity: I^2 = 26% [0%; 51%], τ^2 = 0.0640 [0.0000; 0.0979], p = 0.07

After outlier removal

RoB: Risk Of Bias;

D1- bias arising from the randomisation process

D2- bias due to deviations from intended interventions

D3- bias due to missing outcome data

D4- bias in measurement in outcome

D5- bias in selection of reported result

Figure S9: Risk of bias plot – domain wise

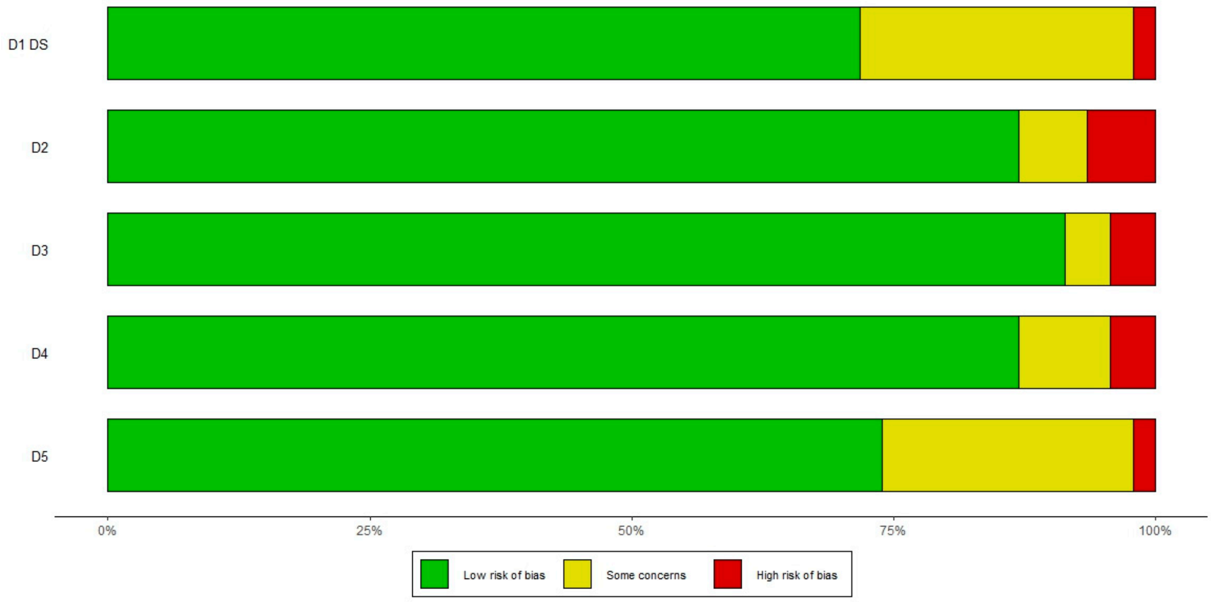

#D1-D5 – domains for RoB assessment in parallel group design studies, DS – additional domain besides D1-D5 in cross over design studies

Figure S10: Funnel plot after outlier removal

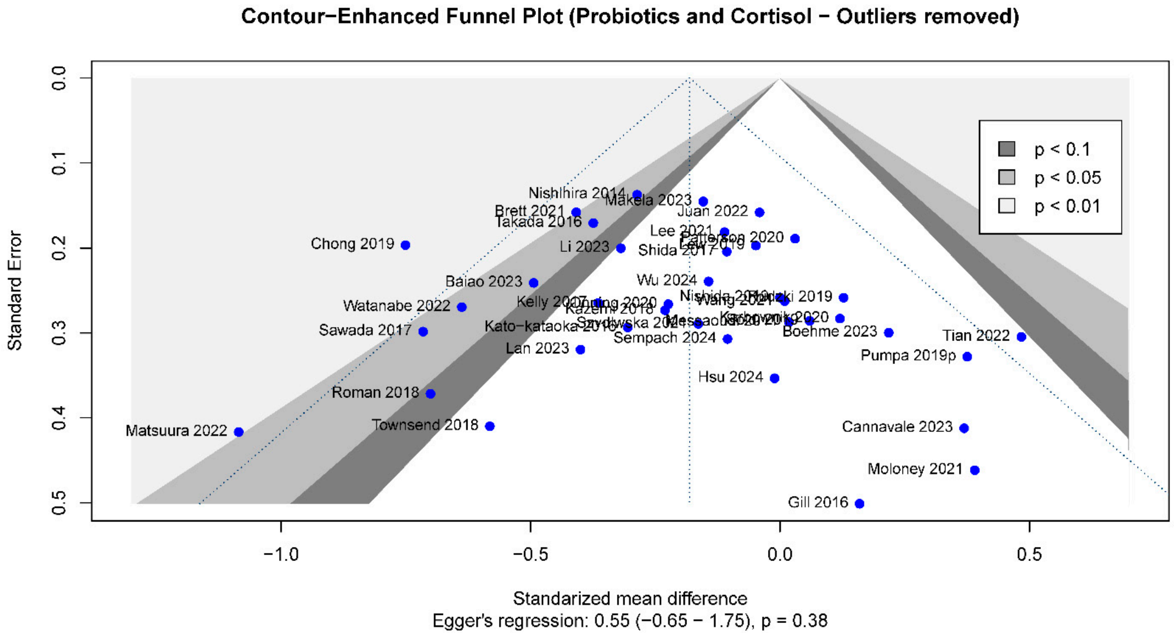

Supplement: Supplementary file 1 [file nutrients-16-03564-s001.zip › supplementary.pdf]
